# The trajectory of clinical responses in patients with early rheumatoid arthritis who achieve sustained remission in response to abatacept: subanalysis of AVERT-2, a randomized phase IIIb study

**DOI:** 10.1186/s13075-023-03038-2

**Published:** 2023-04-22

**Authors:** Paul Emery, Yoshiya Tanaka, Vivian P. Bykerk, Clifton O. Bingham, Thomas W. J. Huizinga, Gustavo Citera, Kuan-Hsiang Gary Huang, Chun Wu, Sean E. Connolly, Yedid Elbez, Robert Wong, Karissa Lozenski, Roy Fleischmann

**Affiliations:** 1 Leeds Institute of Rheumatic and Musculoskeletal Medicine, University of Leeds and Leeds NIHR Biomedical Research Centre, Leeds, UK; 2 University of Occupational and Environmental Health, Japan, Kitakyushu, Japan; 3 Hospital for Special Surgery, New York, NY USA; 4 Johns Hopkins University, Baltimore, MD USA; 5 Leiden University Medical Center, Leiden, the Netherlands; 6 Instituto de Rehabilitación Psicofísica, Buenos Aires, Argentina; 7 Bristol Myers Squibb, Princeton, NJ USA; 8 Signifience, Puteaux, France; 9 University of Texas Southwestern Medical Center, Dallas, TX USA

**Keywords:** Rheumatoid arthritis, Anti-citrullinated protein antibody, Sustained remission, Abatacept

## Abstract

**Background:**

AVERT-2 (a phase IIIb, two-stage study) evaluated abatacept + methotrexate versus methotrexate alone, in methotrexate-naive, anti-citrullinated protein antibody-positive patients with early (≤ 6 months), active RA. This subanalysis investigated whether individual patients who achieved the week 24 Simplified Disease Activity Index (SDAI) remission primary endpoint could sustain remission to 1 year and then maintain it following changes in therapy.

**Methods:**

During the 56-week induction period (IP), patients were randomized to weekly subcutaneous abatacept 125 mg + methotrexate or abatacept placebo + methotrexate. Patients completing the IP who achieved SDAI remission (≤ 3.3) at weeks 40 and 52 entered a 48-week de-escalation (DE) period. Patients treated with abatacept + methotrexate were re-randomized to continue weekly abatacept + methotrexate, or de-escalate and then withdraw abatacept (after 24 weeks), or receive abatacept monotherapy. Proportions of patients achieving sustained SDAI and Boolean remission, and Disease Activity Score in 28 joints using C-reactive protein (DAS28 [CRP]) < 2.6, were assessed. For patients achieving early sustained SDAI remission at weeks 24/40/52, flow between disease activity categories and individual trajectories was evaluated; flow was also evaluated for later remitters (weeks 40/52 but not week 24).

**Results:**

Among patients treated with abatacept + methotrexate (*n*/*N* = 451/752) at IP week 24, 22% achieved SDAI remission, 17% achieved Boolean remission, and 42% achieved DAS28 (CRP) < 2.6; of these, 56%, 58%, and 74%, respectively, sustained a response throughout IP weeks 40/52. Among patients with a sustained response at IP weeks 24/40/52, 82% (14/17) on weekly abatacept + methotrexate, 81% (13/16) on abatacept monotherapy, 63% (12/19) who de-escalated/withdrew abatacept, and 65% (11/17) on abatacept placebo + methotrexate were in SDAI remission at end of the DE period; rates were higher than for later remitters in all arms except abatacept placebo + methotrexate.

**Conclusions:**

A high proportion of individual patients achieving clinical endpoints at IP week 24 with abatacept + methotrexate sustained their responses through week 52. Of patients achieving early and sustained SDAI remission through 52 weeks, numerically more maintained remission during the DE period if weekly abatacept treatment continued.

**Trial registration:**

NCT02504268 (ClinicalTrials.gov), registered July 21, 2015.

## Background

Rheumatoid arthritis (RA) is characterized by significant synovitis and structural joint damage, as well as systemic inflammation and extra-articular manifestations of disease [1, 2]. RA that is left untreated or undertreated may lead to cumulative and irreversible joint damage, impairment of physical function, increased morbidity, and risk of early mortality, particularly cardiovascular- and respiratory-related death [1, 2].

Treating to target is an intensive and dynamic strategy [3, 4] endorsed by both the American College of Rheumatology (ACR) and the European Alliance of Associations for Rheumatology (EULAR) [5–7]. Clinical remission has been considered the main therapeutic goal, with low disease activity (LDA) a reasonable alternative [5–7] if remission cannot be reached, although recent guidance conditionally suggests an initial target of LDA with a subsequent goal of remission [7]. The target, as defined by ACR and EULAR, is sustained reduction in disease activity as measured by the Simplified Disease Activity Index (SDAI; score of ≤ 3.3) or Boolean remission [5–7]. This strategy, together with the broad range of disease-modifying antirheumatic drugs (DMARDs) now available, means that effective disease control may be attainable for many patients with RA [6, 8].

Multiple studies have demonstrated sustained remission or LDA, inhibition of radiographic progression, and reduced physical disability in patients with RA in response to early, aggressive treatment [2, 9–19]. This putative “window of opportunity” in RA suggests the possibility that early, intensive treatment may alter the long-term trajectory of the disease [16, 20]. Both ACR and EULAR guidelines agree that tapering therapy, through reduction of dose or dose frequency, may be achievable in some patients [5, 6], and clinical studies have shown that a small number are able to maintain disease remission or LDA following DMARD tapering [8]. However, drug-free remission is not sustainable in the majority of patients [9, 18, 21–23], and flares after tapering or stopping treatment are associated with progression of joint damage [24, 25].

Currently, treatment decisions are based on group-level efficacy data from clinical trials. However, a better understanding of an individual patient’s long-term response to particular treatment strategies is desirable. It would be useful to know, for example, whether the majority of patients who achieve early (e.g., within 6 months [26]) and durable (e.g., for at least 6 months [26, 27]) remission in response to a particular drug regimen will then maintain that response over the longer term.

The selective co-stimulation modulator abatacept, which disrupts naive T cell activation, is effective in treating patients with early RA [9, 28]. The phase IIIb AVERT (*A*ssessing *V*ery *E*arly *R*A *T*reatment)-2 (NCT02504268) study evaluated the efficacy and safety of subcutaneous (SC) abatacept + methotrexate (MTX) versus abatacept placebo + MTX in biomarker-defined, MTX-naive patients with early, active RA during a 56-week induction period (IP). This was followed by a further 48-week de-escalation (DE) period [29]. Although the primary endpoint of SDAI remission (≤ 3.3) at IP week 24 was not met, at IP week 52, numerically more patients in the primary analysis population of AVERT-2 achieved SDAI remission (≤ 3.3) with abatacept + MTX versus abatacept placebo + MTX [29].

While the primary analysis assessed the overall percentage of patients achieving SDAI remission in a defined subset of the entire study population (primary analysis population; *n* = 375) [29], it is not known whether individual patients within a treatment group achieved and sustained the same stringent and clinically meaningful efficacy outcomes at all time points during the IP and DE period. Such data could aid prescribing clinicians when making treatment decisions at the individual patient level. The subanalysis of the AVERT-2 study reported here investigated whether individual patients who achieved early clinical responses to abatacept according to stringent SDAI criteria (SDAI remission ≤ 3.3 at IP week 24) and sustained that response through IP week 52 were able to maintain SDAI remission during the DE period following changes in therapy.

## Methods

### Study design

**Fig. 1:**
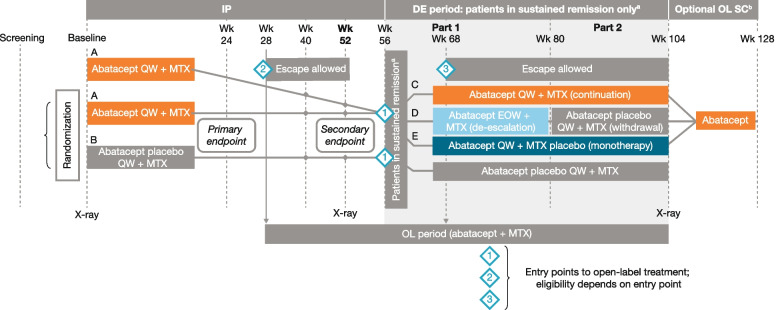
Study design. An IP of 56 weeks was followed by a 48-week DE period for patients in sustained SDAI remission (≤ 3.3 at both weeks 40 and 52 in the IP) and a 24-week post-treatment follow-up period (all patients). DE, de-escalation; EOW, every other week; IP, induction period; MTX, methotrexate; OL, open-label; QW, once-weekly; SC, subcutaneous; SDAI, Simplified Disease Activity Index; Wk, week. ^a^SDAI ≤ 3.3 at both weeks 40 and 52: patients from treatment arm A were re-randomized into the DE period to one of three treatment arms (C: continuation, D: DE followed by withdrawal, or E: monotherapy) in a ratio of 1:1:1 at week 56. Patients in sustained SDAI remission from treatment arm B continued to receive this treatment in a blinded fashion. ^b^DE completers. Previously presented at EULAR 2020 (poster SAT0104); copyright © the authors

Full details of the study design have been published previously [29]. Briefly, AVERT-2 (NCT02504268) was a phase IIIb, 132-week, randomized, double-blind, placebo-controlled study of MTX-naive, anti-citrullinated protein antibody (ACPA)-positive patients with active, early RA. During the IP, patients were randomized (3:2) to once-weekly (QW) SC abatacept 125 mg + MTX (starting dose of 7.5–15 mg/week titrated to ≥ 15 mg, as tolerated, and as per local practice and regulations, within 8 weeks) or abatacept placebo + MTX for 56 weeks (Fig. 1).

Patients who completed the IP and achieved sustained SDAI remission (≤ 3.3 at both weeks 40 and 52) entered a 48-week DE period. Patients entering the DE period who were originally treated with abatacept + MTX were re-randomized (1:1:1) to one of three abatacept treatment arms: continuation (abatacept QW + MTX for 48 weeks); stepwise DE and withdrawal (abatacept every other week [EOW] + MTX for 24 weeks [part 1] followed by abatacept placebo + MTX for 24 weeks [part 2]); or abatacept monotherapy (abatacept QW + MTX placebo for 48 weeks) (Fig. 1). Patients who received abatacept placebo + MTX and attained sustained SDAI remission during the IP continued the same treatment in the DE period in a blinded fashion.

The study was conducted in accordance with the Declaration of Helsinki and International Conference on Harmonisation Good Clinical Practice. The protocol and patients’ informed consent received institutional review board/independent ethics committee approval prior to initiation of the study, and all patients provided informed consent prior to enrollment.

### Patient population

Full inclusion and exclusion criteria have been published previously [29]. Briefly, patients aged ≥ 18 years with a diagnosis of RA (ACR/EULAR 2010 criteria) [30] for ≤ 6 months who were ACPA-positive and DMARD-naive were eligible for the study if they had: a tender joint count (TJC) ≥ 3, a swollen joint count (SJC) ≥ 3, C-reactive protein (CRP) > 3.0 mg/L or erythrocyte sedimentation rate (ESR) ≥ 28 mm/h, and baseline SDAI score > 11. The present analysis was carried out in the intent-to-treat (ITT) population, i.e., all randomized patients who received ≥ 1 dose of the study drug during the 56-week IP.

### Study outcomes and assessments

In this post hoc analysis, the proportions of patients achieving response at IP week 24 and at both IP weeks 40 and 52 (early sustained response) were assessed. The proportion of patients achieving the stringent endpoints of remission according to SDAI and Boolean criteria, and other endpoints, including Disease Activity Score in 28 joints using CRP (DAS28 [CRP]) < 2.6, during the IP were investigated by treatment arm.

The flow of these patients in early sustained remission between SDAI categories (remission, LDA [> 3.3–11], moderate disease activity [> 11–26], and high disease activity [> 26]) during both the IP and DE period were plotted using Sankey diagrams. The Sankey diagram format was used to evaluate patterns of disease activity following de-escalation of abatacept in patients with sustained remission. As a comparison with patients with early sustained SDAI remission (IP weeks 24/40/52), Sankey diagrams were also plotted for patients with later sustained SDAI remission (IP weeks 40/52 but not 24).

Heat maps were used to plot color-coded SDAI data across time points to visualize response trajectories for individual patients with early sustained (weeks 24/40/52) SDAI remission during both the IP and 48-week DE period.

## Results

### Overall ITT population

#### Patient disposition and baseline characteristics

Overall, as reported previously [29], 752 patients were randomized to receive either abatacept + MTX (*n* = 451) or abatacept placebo + MTX (*n* = 301); 63 (14%) and 68 (23%), respectively, had discontinued by week 52. Baseline clinical and demographic characteristics have been described previously and were similar across treatment arms [29]. At baseline, disease duration was 1.2–1.3 months, mean SDAI score was 38.2–39.4, and mean DAS28 (CRP) score was 5.6 across the two treatment arms.

#### Clinical response during the IP

**Fig. 2:**
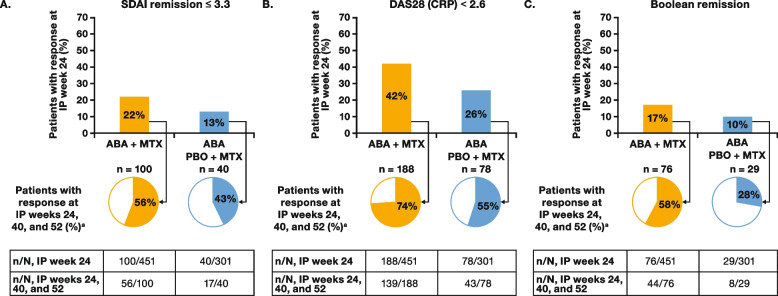
Patients with IP week 24 response who achieved weeks 40 and 52 responses. ABA, abatacept; DAS28 (CRP), Disease Activity Score in 28 joints using C-reactive protein; IP, induction period; MTX, methotrexate; PBO, placebo; SDAI, Simplified Disease Activity Index. ^a^Percentages based on number of patients within each treatment group who achieved response at IP week 24 (denominator). Previously presented at EULAR 2020 (poster FRI0090); copyright © the authors

In the overall ITT population, all endpoints evaluated were achieved and sustained by numerically more patients treated with abatacept + MTX versus those treated with abatacept placebo + MTX. SDAI remission at IP week 24 was achieved by 22% (100/451) of patients receiving abatacept + MTX and 13% (40/301) receiving abatacept placebo + MTX, and of these patients, 56% (56/100) and 43% (17/40), respectively, also achieved sustained SDAI remission at IP weeks 40 and 52 (Fig. 2A); these patients formed the early and sustained SDAI remission subpopulation. Similarly, at IP week 24, 42% (188/451) of patients receiving abatacept + MTX and 26% (78/301) receiving abatacept placebo + MTX achieved DAS28 (CRP) < 2.6, of whom 74% (139/188) and 55% (43/78), respectively, had sustained DAS28 (CRP) < 2.6 at IP weeks 24, 40, and 52 (Fig. 2B). Similar patterns of sustained response were observed for Boolean remission in both treatment arms. Consistent with the above, 17% (76/451) of patients receiving abatacept + MTX and 10% (29/301) receiving abatacept placebo + MTX achieved Boolean remission at IP week 24, of whom 58% (44/76) and 28% (8/29), respectively, had sustained Boolean remission at IP weeks 40 and 52 (Fig. 2C).

#### Patient flow through SDAI categories during the IP

**Fig. 3:**
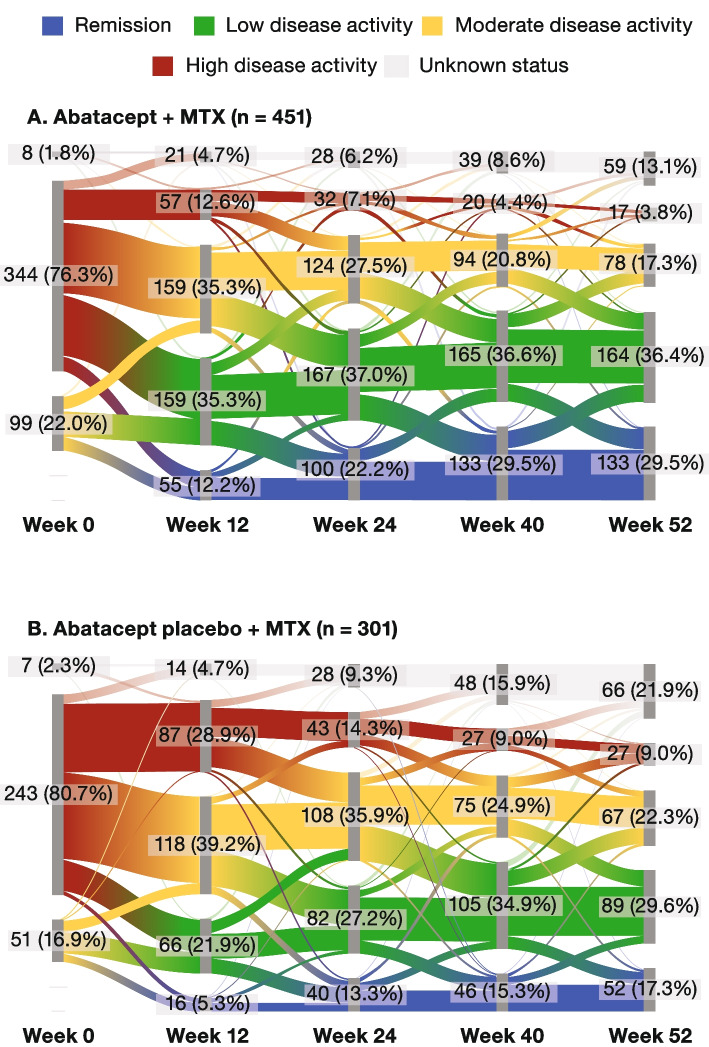
Summary of SDAI categories over time in the intent-to-treat population during the induction period. Unknown status refers to the patients for whom data were not available at that particular time point. SDAI categories: remission, ≤ 3.3; low disease activity, > 3.3–11; moderate disease activity, > 11–26; high disease activity, > 26. MTX, methotrexate; SDAI, Simplified Disease Activity Index. Portions of panel **A** were previously presented at EULAR 2020 (poster FRI0090); copyright © the authors

The flow of patients through the SDAI categories during the IP is shown in Fig. 3, with the width of each ribbon based on the proportion of patients represented. Over time, numerically more patients in the abatacept + MTX treatment arm were in a state of SDAI remission (Fig. 3A) or LDA than patients who received treatment with abatacept placebo + MTX (Fig. 3B). Although all patients had achieved SDAI remission by week 24, improvements in disease activity states also occurred earlier for patients in the abatacept + MTX treatment arm: at week 12 of the IP, 55 (12%) and 159 (35%) of patients receiving abatacept + MTX were in SDAI remission or LDA, respectively, compared with 16 (5%) and 66 (22%) of patients receiving abatacept placebo + MTX (Fig. 3).

### Early and sustained SDAI remission subpopulation

#### Patient disposition and baseline characteristics

Overall, 56 patients in the abatacept + MTX arm and 17 patients in the abatacept placebo + MTX arm achieved early sustained SDAI remission during the IP (Table 1). Baseline demographics and RA disease duration were similar between the abatacept + MTX and abatacept placebo + MTX groups in this subpopulation (Table 1).

**Table 1:** Baseline demographics and disease characteristics of patients by sustained SDAI remission status^a^

**Characteristic**	**Patients who achieved sustained SDAI remission**^**a**^		**Patients who did not achieve sustained SDAI remission**^**a**^	
	**Abatacept + MTX** **(*n* = 56)**	**Abatacept placebo + MTX** **(*n* = 17)**	**Abatacept + MTX** **(*n* = 395)**	**Abatacept placebo + MTX** **(*n* = 284)**
Age, years	44.9 (13.8)	41.3 (13.1)	49.5 (12.5)	49.2 (13.9)
Female sex, *n* (%)	39 (69.6)	13 (76.5)	310 (78.5)	230 (81.0)
Race, *n* (%)				
White	43 (76.8)	13 (76.5)	272 (68.9)	196 (69.0)
Asian	9 (16.1)	4 (23.5)	68 (17.2)	48 (16.9)
Black/African American	0	0	20 (5.1)	16 (5.6)
American Indian/Alaska Native	0	0	0	3 (1.1)
Other	4 (7.1)	0	35 (8.9)	21 (7.4)
Geographic region, *n* (%)				
North America	2 (3.6)	0	57 (14.4)	47 (16.5)
South America	30 (53.6)	9 (52.9)	163 (41.3)	103 (36.3)
Asia	9 (16.1)	4 (23.5)	67 (17.0)	46 (16.2)
Europe	14 (25.0)	4 (23.5)	84 (21.3)	64 (22.5)
Rest of world	1 (1.8)	0	24 (6.1)	24 (8.5)
RA disease duration, months	1.0 (1.2)	1.2 (1.5)	1.2 (1.4)	1.3 (1.4)
RF positive, *n* (%)	53 (94.6)	14 (82.4)	367 (92.9)	265 (93.3)
Tender joint count (28 joints)	10.1 (5.1)	8.6 (4.0)	13.6 (6.9)	14.0 (6.8)
Swollen joint count (28 joints)	8.1 (4.4)	7.5 (2.6)	10.3 (5.8)	10.9 (5.9)
CRP, mg/L	11.4 (14.1)	9.8 (9.5)	21.2 (28.2)	19.6 (22.4)
Patient Global Assessment of disease activity	65.2 (19.6)	52.2 (22.1)	65.7 (23.1)	63.4 (24.1)
Physician Global Assessment of disease activity	63.1 (16.2)	47.5 (13.2)	65.4 (18.8)	67.2 (19.5)
SDAI	32.18 (10.5)	27.12 (8.1)	39.1 (14.3)	40.1 (13.7)
DAS28 (CRP)	5.1 (0.8)	4.8 (0.9)	5.6 (1.1)	5.7 (1.0)
HAQ-DI	1.4 (0.8)	1.2 (0.8)	1.6 (0.7)	1.6 (0.7)
Patient assessment of pain (0–100 mm VAS)	65.9 (18.5)	54.1 (25.5)	66.6 (23.1)	66.1 (22.1)

Data are presented as mean (SD), unless stated otherwise

*CRP* C-reactive protein, *DAS28 (CRP)* Disease Activity Score in 28 joints using C-reactive protein, *HAQ-DI* Health Assessment Questionnaire-Disability Index, *MTX* methotrexate, *RA* rheumatoid arthritis, *RF* rheumatoid factor, *SD* standard deviation, *SDAI* Simplified Disease Activity Index, *VAS* visual analog scale

^a^At weeks 24, 40, and 52 of the study induction period

At baseline, rheumatoid factor positivity, TJC/SJC in 28 joints (TJC28/SJC28), CRP, Patient and Physician Global Assessments of disease activity, SDAI, DAS28 (CRP), Health Assessment Questionnaire-Disability Index (HAQ-DI), and patients’ assessment of pain were all slightly numerically higher in the abatacept + MTX arm versus the abatacept placebo + MTX arm, although these differences are unlikely to be clinically meaningful. By comparison, in those patients who did not achieve sustained SDAI remission, baseline demographics and disease characteristics were also similar between the abatacept + MTX and abatacept placebo + MTX groups (Table 1). There were some differences between the subgroups of patients who did or did not achieve sustained SDAI remission, with patients who did not achieve remission being older, more likely to be female, and having higher baseline disease activity (SDAI and DAS28 [CRP]) than those who did achieve remission (Table 1).

Compared with the overall ITT population [29], lower baseline mean scores for TJC28/SJC28, SDAI, and HAQ-DI were observed among patients who achieved early sustained SDAI remission in both treatment arms. Lower mean scores for Patient and Physician Global Assessments of disease activity and patients’ assessment of pain were also observed for the abatacept placebo + MTX group.

#### Patient flow through SDAI categories during the DE period

**Fig. 4:**
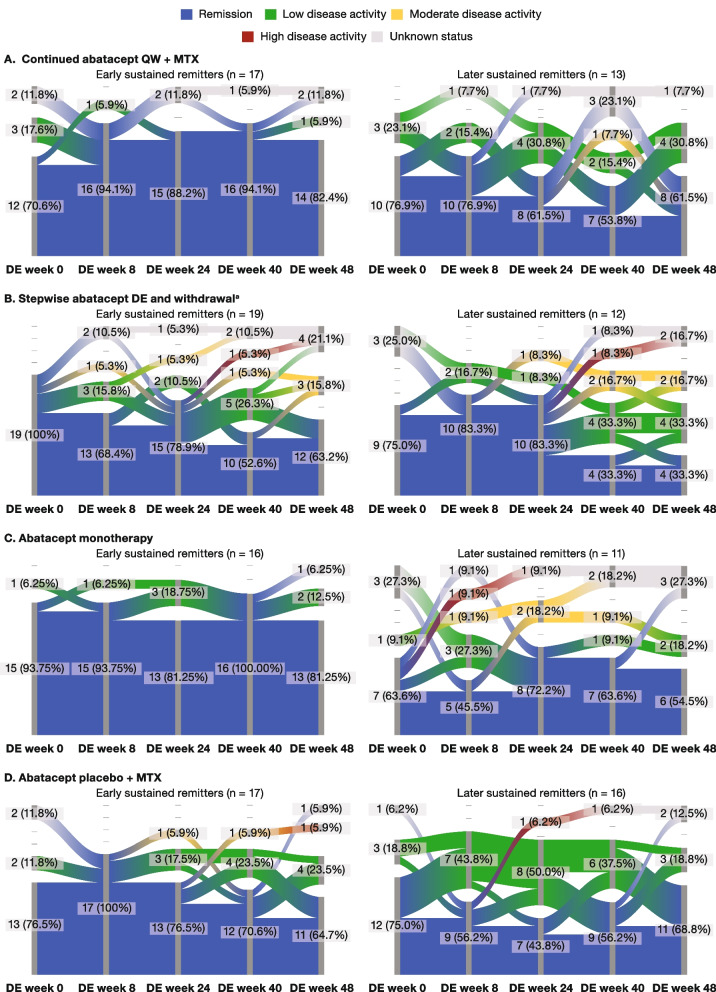
DE-period SDAI categories in patients achieving sustained SDAI remission early^b^ or later^c^ during the IP; intent-to-treat population. Unknown status refers to the patients for whom data were not available at that particular time point. SDAI categories: remission, ≤ 3.3; low disease activity, > 3.3–11; moderate disease activity, > 11–26; high disease activity, > 26. For parts A, B, and C, patients received SC abatacept QW + MTX during the IP^d^. For part D, patients received abatacept placebo + MTX during the IP and continued the same randomized treatment in the DE period. DE, de-escalation; EOW, every other week; IP, induction period; MTX, methotrexate; QW, once-weekly; SC, subcutaneous; SDAI, Simplified Disease Activity Index. ^a^Part 1: abatacept EOW + MTX for 24 weeks; part 2: abatacept placebo + MTX for 24 weeks. ^b^IP weeks 24, 40, and 52. ^c^IP weeks 40 and 52, not 24. ^d^Of the 56 patients from the abatacept + MTX arm who achieved sustained SDAI remission at weeks 24, 40, and 52 during the IP, SDAI status was unknown for four patients during the DE period of the study (data not shown)

Of the 56 patients from the abatacept + MTX arm who achieved early (at week 24) sustained SDAI remission during the IP, SDAI status was unknown for four patients during the DE period of the study (data not shown). For the remaining patients who achieved early sustained SDAI remission, the flow of the population through SDAI categories during the DE period is shown in Fig. 4. In addition, flow through SDAI categories is also shown for the patients who achieved later sustained SDAI remission at IP weeks 40 and 52 but not 24 (later sustained remitters).

Among patients who achieved early sustained remission, a higher proportion who were re-randomized to continue abatacept QW + MTX (82% [14/17]) or receive abatacept monotherapy (81% [13/16]) at week 56 were in remission at the end of the DE phase (DE week 48) compared with patients who were re-randomized to de-escalate to abatacept EOW + MTX for 24 weeks and then stop abatacept treatment (63% [12/19]) (Fig. 4A–C, left-hand panels). Although patient numbers per group were small, there was also less movement between SDAI categories in patients who continued abatacept with MTX or as monotherapy compared with the abatacept de-escalation/withdrawal arm (Fig. 4A–C, left-hand panels). Among patients who were randomized to de-escalate and then stop abatacept treatment, patients lost SDAI remission status more frequently in part 2 of the DE period after they had stopped abatacept treatment completely (Fig. 4B); this was not seen in patients who continued abatacept with MTX (Fig. 4A) or as monotherapy (Fig. 4C). In all treatment groups except abatacept placebo + MTX, remission rates at DE week 48 for early sustained remitters were higher than for later sustained remitters (in remission at IP weeks 40 and 52 but not 24; Fig. 4A–C, left-hand vs right-hand panels); there was also generally less movement between SDAI categories for early sustained remitters compared with later sustained remitters.

#### Individual patient SDAI responses during the DE period

**Fig. 5:**
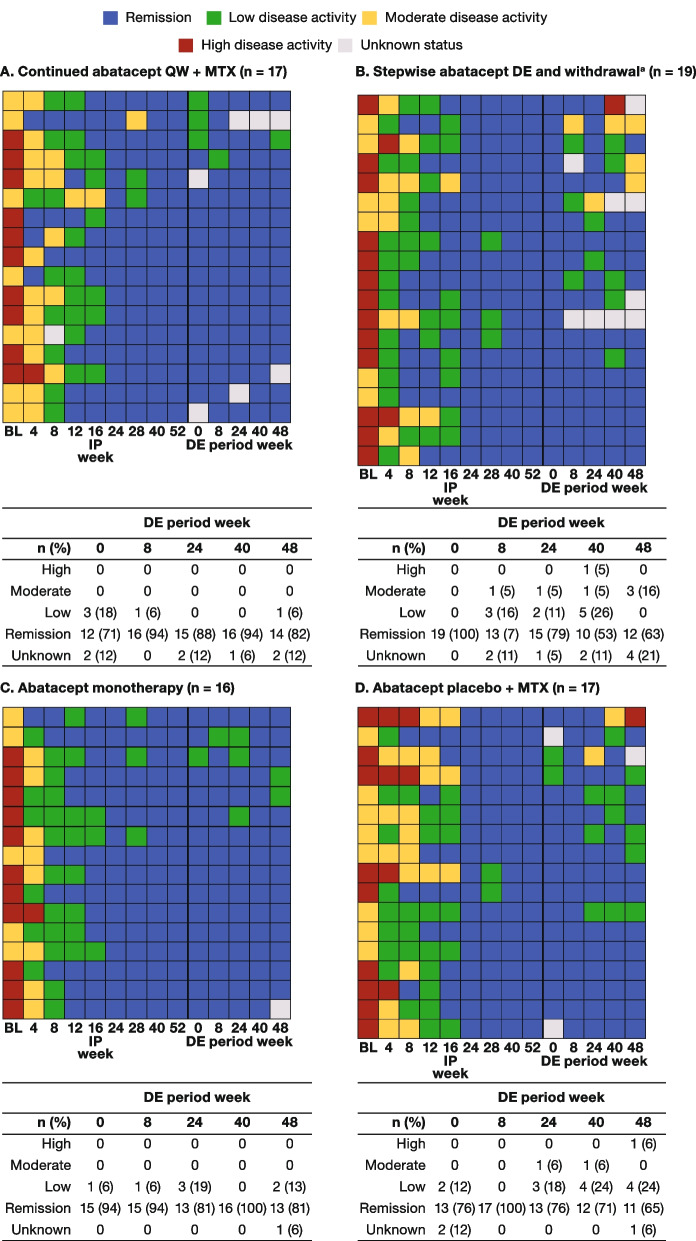
Individual SDAI responses (patients achieving early [IP week 24] sustained [through IP week 52^b^] SDAI remission). Each row represents an individual patient in the intent-to-treat population. For parts A, B, and C, patients received SC abatacept QW + MTX during the IP^c^. For part D, patients received abatacept placebo + MTX during the IP and continued the same randomized treatment in the DE period. BL, baseline; DE, de-escalation; EOW, every other week; IP, induction period; MTX, methotrexate; QW, once-weekly; SC, subcutaneous; SDAI, Simplified Disease Activity Index. ^a^Part 1: abatacept EOW + MTX for 24 weeks; part 2: abatacept placebo + MTX for 24 weeks. ^b^Defined as remission at both weeks 40 and 52. ^c^Of the 56 patients from the abatacept + MTX arm who achieved sustained SDAI remission at weeks 24, 40, and 52 during the IP, SDAI status was unknown for four patients during the DE period of the study (data not shown)

In the subpopulation of patients who achieved early (at week 24) sustained SDAI remission, individual patient trajectory data presented as heatmaps show that more patients maintained SDAI remission at all study visits during the DE period if they continued abatacept (either abatacept + MTX, 53% [9/17] or abatacept monotherapy, 63% [10/16]) than if they de-escalated/withdrew abatacept (37% [7/19]) or received abatacept placebo + MTX (41% [7/17]; Fig. 5A–D). Of the patients who achieved sustained remission during the IP on abatacept placebo + MTX therapy and continued with the same regimen during the DE period, 65% (11/17) were in SDAI remission at the end of the de-escalation phase (Fig. 4D).

## Discussion

In this subanalysis of a phase IIIb study in ACPA-positive patients with early RA, a higher number of individual patients who received SC abatacept QW + MTX achieved clinically stringent (SDAI and Boolean remission) endpoints early at IP week 24 and sustained these responses through IP week 52 than patients who initially received abatacept placebo + MTX. Furthermore, of the patients who achieved early sustained SDAI remission through IP week 52, numerically more of those who continued abatacept QW + MTX during the DE period maintained SDAI remission at further follow-up visits than patients who tapered and then withdrew abatacept therapy or those who continued treatment with abatacept placebo + MTX.

As previously reported for the overall population in the AVERT-2 study, patients who continued abatacept + MTX during the DE period demonstrated better maintenance of SDAI remission and inhibition of structural damage than patients who de-escalated/withdrew abatacept or who continued with abatacept monotherapy [29]. However, such group-level data may not reflect the fact that the same individual patients can achieve and then lose response (and vice versa) during the study; hence, an individual response may not be sustained. The present subanalysis of AVERT-2 assessed individual patients who achieved early SDAI remission and sustained it through IP week 52, and similar findings to the primary analysis of the overall population were observed. Although some individual patients moved in and out of different disease activity levels, a higher number of patients who continued with abatacept + MTX (82%) or who stopped MTX and continued treatment with abatacept monotherapy (81%) remained in SDAI remission at the end of the 48-week DE period compared with patients who underwent stepwise DE (abatacept EOW + MTX for 24 weeks) then withdrawal of abatacept (abatacept placebo + MTX for 24 weeks; 63%) or those who continued abatacept placebo + MTX (65%). Of note, remission rates for early sustained remitters were higher than for later sustained remitters in all arms except abatacept placebo + MTX. This may suggest that early sustained remitter status may be an independent favorable predictor of sustained remission for patients continuing abatacept-containing regimens.

The ability of individual patients to achieve a deeper level of remission, that is both early in the course of a particular treatment regimen and sustained over time, should be beneficial in terms of future joint function and control of inflammation, potentially resulting in less systemic complications of disease. Being able to determine relatively early for each individual patient whether a particular drug or regimen is likely to lead to sustained remission would allow clinicians to make more informed treatment choices, including whether additions or changes to treatment are required to achieve better long-term outcomes. Previous studies have suggested that early improvement in disease activity in response to treatment predicts longer-term outcomes. For example, pooled patient data from early RA clinical trials of MTX, tumor necrosis factor inhibitor (TNFi) monotherapy, or TNFi + MTX combination therapy demonstrated significant correlation between disease activity (SDAI) during the first 3 months of treatment and disease activity after 1 year of therapy [3]. In another study of biologic DMARD-naive patients with RA initiating a TNFi, DAS28 (ESR) scores at 6 months were predictive of long-term outcomes [31]. Although patients without early disease control can achieve favorable clinical and functional outcomes when switched to a different regimen [17, 24, 32–35], early and sustained disease control is important to achieving favorable long-term radiographic outcomes and reduction in physical disability before significant deterioration occurs. Further research is needed to understand the impact of early and sustained disease control on radiographic outcomes at an individual level. For example, a study of registry data showed that in patients with early RA treated using a treat-to-target strategy, radiographic progression appears to be a disease process that is individually determined and driven by multiple factors [36]. Continued characterization of individual patients who achieve and sustain a treatment response will enable better prediction of which patients will respond.

Following the advent of treat-to-target and tight control strategies for the management of RA, together with the wide availability of biologic/targeted synthetic DMARDs, RA is now a disease that can be controlled in many patients [8]. However, whether some patients whose RA has been controlled can successfully stop treatment remains to be fully determined. Reports on the number of patients who sustain remission/LDA following de-escalation vary widely [8, 23, 37–46]; it is difficult and inappropriate to make direct comparisons across studies due to differences in patient populations and study designs. It seems apparent, however, that complete drug-free remission is achievable only in a small proportion of patients [9, 14, 23, 39]. In addition, while some patients can successfully sustain remission/LDA following dose tapering or stopping biologic/targeted synthetic DMARDs but retaining background MTX, many will experience worsening of disease state when tapering therapy [8, 23, 37–42, 44–47]. To make informed decisions for optimal patient health, we need more insight to understand which groups of patients will benefit from treatment withdrawal/tapering over the long term and to identify factors that predict successful de-escalation to guide treatment management decisions for individual patients.

Potential indicators of patients suitable for successful tapering of other DMARDs have been investigated previously. For example, in a post hoc analysis of data from three clinical trials, among patients with moderate-to-severe RA, those who achieved sustained Boolean or Clinical Disease Activity Index remission or DAS28 (CRP) < 2.6 and/or “deep remission” (DAS28 [CRP] ≤ 1.98) were more likely to maintain remission/LDA after etanercept dose reduction/withdrawal than patients who only achieved remission or LDA [37]. Previous studies have largely used either DAS28 (CRP or ESR) < 2.6 or ≤ 3.2 as inclusion criteria [8]. To the best of our knowledge, the present study is the first to assess individual patients’ ability to maintain the more stringent outcome of SDAI remission (≤ 3.3) following tapering/withdrawal of a biologic DMARD or MTX. It remains to be determined whether more stringent definitions of remission (SDAI, Boolean) or cut-offs for DAS28 (≤ 1.98, ≤ 1.61) would be more appropriate criteria for determining which patients are suitable for de-escalation of therapy.

There were several strengths and limitations to the AVERT-2 study and this subsequent post hoc analysis. This is the first study to use stringent sustained SDAI remission (≤ 3.3 at both weeks 40 and 52) as a criterion for de-escalation/withdrawal, and de-escalation/withdrawal strategies were assessed in a relatively large population of patients (*n* = 752) compared with many previous de-escalation trials. However, as a post hoc analysis of a randomized, placebo-controlled clinical trial, this study was not powered to show a statistically significant difference between treatment arms for individual patients who achieved and sustained a clinical response in any of the outcomes presented. In addition, patients were required to have an early and sustained stringent response of SDAI ≤ 3.3 at IP weeks 24, 40, and 52 to be included in this subanalysis; thus, the numbers of patients were relatively small, and the findings would need to be verified in a larger population. The AVERT-2 study included a select group of patients with very early (≤ 6 months duration) biomarker-defined (ACPA-positive) RA, and as such, the results may not be generalizable to other RA populations (e.g., those with more advanced RA or seronegative disease). In addition, the follow-up in the DE period subsequent to treatment de-escalation/withdrawal was relatively short (48 weeks), and the outcomes of such de-escalation/withdrawal strategies over the longer term need to be determined.

## Conclusions

In this post hoc evaluation of AVERT-2, among individual patients with early, biomarker-defined RA who achieved clinically stringent endpoints such as SDAI or Boolean remission at IP week 24 with SC abatacept QW + MTX, a high proportion of individual patients (56–58%) sustained their responses through week 52.

Furthermore, numerically more patients who achieved early and sustained SDAI remission in the IP maintained remission during the DE period of the study if they continued weekly treatment with abatacept (either in combination with MTX or as monotherapy) than patients who tapered then withdrew abatacept therapy or those who continued treatment with abatacept placebo + MTX. The achievement of early remission or other clinically relevant outcomes by individual patients treated with weekly SC abatacept + MTX may be indicative of sustained efficacy over time.

## Data Availability

Bristol Myers Squibb policy on data sharing may be found at https://www.bms.com/researchers-and-partners/independent-research/data-sharing-request-process.html.

## Abbreviations

ABA: Abatacept
ACPA: Anti-citrullinated protein antibody
ACR: American College of Rheumatology
AVERT: Assessing Very Early RA Treatment
BL: Baseline
CRP: C-reactive protein
DAS28: Disease Activity Score in 28 joints
DE: De-escalation
DMARD: Disease-modifying antirheumatic drug
EOW: Every other week
ESR: Erythrocyte sedimentation rate
EULAR: European Alliance of Associations for Rheumatology
HAQ-DI: Health Assessment Questionnaire-Disability Index
HDA: High disease activity
IP: Induction period
ITT: Intent-to-treat
LDA: Low disease activity
MDA: Moderate disease activity
MTX: Methotrexate
OL: Open-label
PBO: Placebo
QW: Once-weekly
RA: Rheumatoid arthritis
RF: Rheumatoid factor
SC: Subcutaneous
SD: Standard deviation
SDAI: Simplified Disease Activity Index
SJC: Swollen joint count
TJC: Tender joint count
TNFi: Tumor necrosis factor inhibitor
VAS: Visual analog scale
wk: Week
